# *N*-(1,5-Dimethyl-3-oxo-2-phenyl-2,3-dihydro-1*H*-pyrazol-4-yl)-2-[4-(methyl­sulfan­yl)phen­yl]acetamide

**DOI:** 10.1107/S1600536812034605

**Published:** 2012-08-11

**Authors:** Hoong-Kun Fun, Ching Kheng Quah, Prakash S. Nayak, B. Narayana, B. K. Sarojini

**Affiliations:** a X-ray Crystallography Unit, School of Physics, Universiti Sains Malaysia, 11800 USM, Penang, Malaysia; b Department of Studies in Chemistry, Mangalore University, Mangalagangotri 574 199, India; c Department of Chemistry, P. A. College of Engineering, Nadupadavu, Mangalore 574 153, India

## Abstract

In the title compound, C_20_H_21_N_3_O_2_S, the 2,3-dihydro-1*H*-pyrazole ring is nearly planar (r.m.s. deviation = 0.023 Å) and forms dihedral angles of 16.96 (6) and 38.93 (6)° with the benzene and phenyl rings, respectively. The dihedral angle between the benzene and phenyl rings is 55.54 (6)°. The mol­ecular conformation is consolidated by an intra­molecular C—H⋯O hydrogen bond, which forms an *S*(6) ring. In the crystal, inversion dimers linked by pairs of N—H⋯O_p_ (p = pyrazole) hydrogen bonds generate *R*_2_^2^(10) loops. The dimers are linked by C—H⋯O hydrogen bonds into sheets lying parallel to (100).

## Related literature

For general background to the title compound and for related structures, see: Fun *et al.* (2011*a* ▶,*b* ▶, 2012*a* ▶,*b* ▶). For the stability of the temperature controller used in the the data collection, see: Cosier & Glazer (1986 ▶). For hydrogen-bond motifs, see: Bernstein *et al.* (1995 ▶).
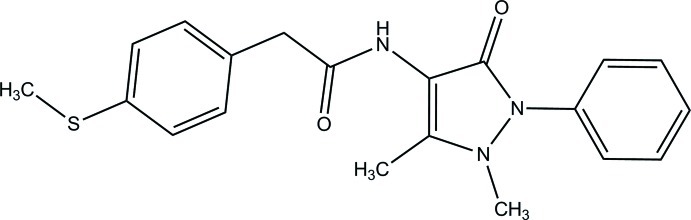


## Experimental

### 

#### Crystal data

C_20_H_21_N_3_O_2_S
*M* *_r_* = 367.46
Monoclinic, 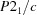

*a* = 14.9176 (8) Å
*b* = 6.6527 (4) Å
*c* = 19.5792 (10) Å
β = 110.689 (1)°
*V* = 1817.78 (17) Å^3^
*Z* = 4
Mo *K*α radiation
μ = 0.20 mm^−1^
*T* = 100 K
0.37 × 0.18 × 0.07 mm

#### Data collection

Bruker SMART APEXII DUO CCD diffractometer
Absorption correction: multi-scan (*SADABS*; Bruker, 2009 ▶) *T*_min_ = 0.931, *T*_max_ = 0.987
19714 measured reflections
5302 independent reflections
4231 reflections with *I* > 2σ(*I*)
*R*_int_ = 0.033

#### Refinement

*R*[*F* ^2^ > 2σ(*F* ^2^)] = 0.042
*wR*(*F* ^2^) = 0.116
*S* = 1.03
5302 reflections
242 parameters
H atoms treated by a mixture of independent and constrained refinement
Δρ_max_ = 0.42 e Å^−3^
Δρ_min_ = −0.52 e Å^−3^

### 

Data collection: *APEX2* (Bruker, 2009 ▶); cell refinement: *SAINT* (Bruker, 2009 ▶); data reduction: *SAINT*; program(s) used to solve structure: *SHELXTL* (Sheldrick, 2008 ▶); program(s) used to refine structure: *SHELXTL*; molecular graphics: *SHELXTL*; software used to prepare material for publication: *SHELXTL* *PLATON* (Spek, 2009 ▶).

## Supplementary Material

Crystal structure: contains datablock(s) global, I. DOI: 10.1107/S1600536812034605/hb6925sup1.cif

Structure factors: contains datablock(s) I. DOI: 10.1107/S1600536812034605/hb6925Isup2.hkl

Supplementary material file. DOI: 10.1107/S1600536812034605/hb6925Isup3.cml

Additional supplementary materials:  crystallographic information; 3D view; checkCIF report

## Figures and Tables

**Table 1:** Hydrogen-bond geometry (Å, °)

*D*—H⋯*A*	*D*—H	H⋯*A*	*D*⋯*A*	*D*—H⋯*A*
N1—H1*N*1⋯O2^i^	0.880 (19)	1.956 (19)	2.7816 (14)	155.7 (19)
C1—H1*A*⋯O1	0.95	2.38	3.0185 (16)	124
C1—H1*A*⋯O1^ii^	0.95	2.51	3.2342 (16)	133
C7—H7*A*⋯O1^iii^	0.99	2.57	3.4960 (16)	155
C19—H19*B*⋯O2^iv^	0.98	2.55	3.4470 (17)	152

Symmetry codes: (i) 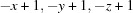; (ii) 
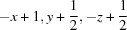
; (iii) 
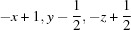
; (iv) 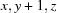.

**Fig. 1.**
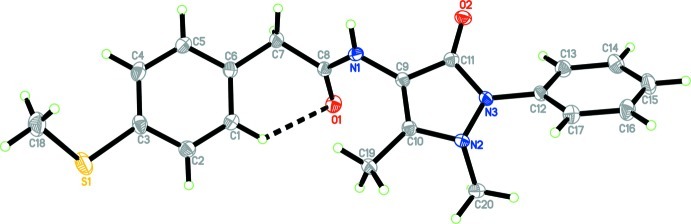
The molecular structure of the title compound showing 50% probability displacement ellipsoids for non-H atoms. Intramolecular hydrogen bond is shown as dashed line.

**Fig. 2.**
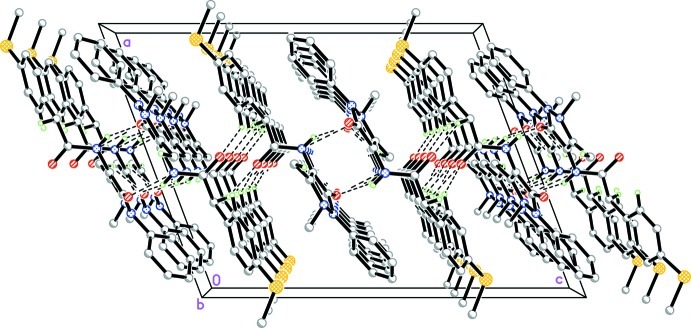
The crystal structure of the title compound, viewed along the b axis. H atoms not involved in hydrogen bonds (dashed lines) have been omitted for clarity.

## supplementary crystallographic information

### Comment

In continuation of our work on synthesis of amides (Fun *et al.*, 2011*a*, 2011*b*, 2012*a*, 2012*b*), we report herein the crystal structure of the title compound.

In the title molecule (Fig. 1), the 1*H*-pyrazol-4-yl ring (N2/N3/C9-C11) is nearly planar (r.m.s. deviation = 0.023 Å) and it forms dihedral angles of 16.96 (6) and 38.93 (6)° with the benzene (C1-C6) and phenyl (C12-C17) rings, respectively. The dihedral angle between the benzene and phenyl rings is 55.54 (6)°. Bond lengths and angles are within normal ranges and are comparable to related structures (Fun *et al.*, 2011*a*, 2011*b*, 2012*a*, 2012*b*). The molecular structure is stabilized by intramolecular C1–H1A···O1 hydrogen bond, forming an S(6) ring motif (Bernstein *et al.*, 1995).

In the crystal structure, Fig. 2, molecules are linked *via* N1–H1N1···O2, C1–H1A···O1, C7–H7A···O1 and C19–H19B···O2 hydrogen bonds (Table 1) into two-dimensional plane parallel to (100) which contains R_2_^2^ (10) ring motifs (Bernstein *et al.*, 1995).

### Experimental

[4-(Methylsulfanyl)phenyl]acetic acid (0.182 g, 1 mmol), 4-amino antipyrine (0.205 g, 1 mmol) and 1-ethyl-3-(3-dimethylaminopropyl)-carbodiimide hydrochloride (1.0 g, 0.01 mol) were dissolved in dichloromethane (20 ml). The mixture was stirred in presence of triethylamine at 273 K for about 3 h. The contents were poured into 100 ml of ice-cold aqueous hydrochloric acid with stirring. The concoction was extracted thrice with dichloromethane. The organic layer was washed with saturated NaHCO_3_ solution and brine solution, dried and concentrated under reduced pressure to give the title compound (I). Yellow plates were grown from an acetone and toluene (1:1) solvent mixture by the slow evaporation method (*m.p.*: 415-418 K).

### Refinement

Atom H1N1 was located in a difference Fourier map and refined freely [N–H = 0.878 (19) Å]. The remaining H atoms were positioned geometrically and refined using a riding model with C–H = 0.95-0.99 Å and *U*_iso_(H) = 1.2 or 1.5 *U*_eq_(C). A rotating-group model was applied for the methyl groups.

### Crystal data

**Table:**

C_20_H_21_N_3_O_2_S	*F*(000) = 776
*M**_r_* = 367.46	*D*_x_ = 1.343 Mg m^−^^3^
Monoclinic, *P*2_1_/*c*	Mo *K*α radiation, λ = 0.71073 Å
Hall symbol: -P 2ybc	Cell parameters from 5722 reflections
*a* = 14.9176 (8) Å	θ = 2.9–30.0°
*b* = 6.6527 (4) Å	µ = 0.20 mm^−^^1^
*c* = 19.5792 (10) Å	*T* = 100 K
β = 110.689 (1)°	Plate, yellow
*V* = 1817.78 (17) Å^3^	0.37 × 0.18 × 0.07 mm
*Z* = 4	

### Data collection

**Table:**

Bruker SMART APEXII DUO CCD diffractometer	5302 independent reflections
Radiation source: fine-focus sealed tube	4231 reflections with *I* > 2σ(*I*)
Graphite monochromator	*R*_int_ = 0.033
φ and ω scans	θ_max_ = 30.1°, θ_min_ = 2.2°
Absorption correction: multi-scan (*SADABS*; Bruker, 2009)	*h* = −20→21
*T*_min_ = 0.931, *T*_max_ = 0.987	*k* = −9→9
19714 measured reflections	*l* = −27→27

### Refinement

**Table:**

Refinement on *F*^2^	Primary atom site location: structure-invariant direct methods
Least-squares matrix: full	Secondary atom site location: difference Fourier map
*R*[*F*^2^ > 2σ(*F*^2^)] = 0.042	Hydrogen site location: inferred from neighbouring sites
*wR*(*F*^2^) = 0.116	H atoms treated by a mixture of independent and constrained refinement
*S* = 1.03	*w* = 1/[σ^2^(*F*_o_^2^) + (0.058*P*)^2^ + 0.594*P*] where *P* = (*F*_o_^2^ + 2*F*_c_^2^)/3
5302 reflections	(Δ/σ)_max_ = 0.001
242 parameters	Δρ_max_ = 0.42 e Å^−^^3^
0 restraints	Δρ_min_ = −0.52 e Å^−^^3^

### Special details

**Table:**

Experimental. The crystal was placed in the cold stream of an Oxford Cryosystems Cobra open-flow nitrogen cryostat (Cosier & Glazer, 1986) operating at 100.0 (1) K.
Geometry. All esds (except the esd in the dihedral angle between two l.s. planes) are estimated using the full covariance matrix. The cell esds are taken into account individually in the estimation of esds in distances, angles and torsion angles; correlations between esds in cell parameters are only used when they are defined by crystal symmetry. An approximate (isotropic) treatment of cell esds is used for estimating esds involving l.s. planes.
Refinement. Refinement of F^2^ against ALL reflections. The weighted R-factor wR and goodness of fit S are based on F^2^, conventional R-factors R are based on F, with F set to zero for negative F^2^. The threshold expression of F^2^ > 2sigma(F^2^) is used only for calculating R-factors(gt) etc. and is not relevant to the choice of reflections for refinement. R-factors based on F^2^ are statistically about twice as large as those based on F, and R- factors based on ALL data will be even larger.

### Fractional atomic coordinates and isotropic or equivalent isotropic displacement parameters (Å^2^)

**Table:**

	*x*	*y*	*z*	*U*_iso_*/*U*_eq_	
S1	0.90518 (2)	1.11552 (8)	0.26045 (2)	0.03908 (13)	
O1	0.48783 (6)	0.70169 (15)	0.28728 (5)	0.0201 (2)	
O2	0.37745 (6)	0.49368 (14)	0.45147 (5)	0.01838 (19)	
N1	0.54078 (7)	0.67806 (17)	0.41084 (6)	0.0162 (2)	
N2	0.35044 (7)	1.00817 (16)	0.41536 (5)	0.0153 (2)	
N3	0.31713 (7)	0.81906 (16)	0.42879 (5)	0.0155 (2)	
C1	0.67740 (8)	0.8798 (2)	0.29532 (7)	0.0182 (2)	
H1A	0.6135	0.9222	0.2866	0.022*	
C2	0.73958 (9)	1.0064 (2)	0.27714 (7)	0.0201 (3)	
H2A	0.7178	1.1344	0.2564	0.024*	
C3	0.83366 (8)	0.9474 (2)	0.28906 (6)	0.0210 (3)	
C4	0.86425 (8)	0.7580 (2)	0.31933 (6)	0.0215 (3)	
H4A	0.9278	0.7148	0.3271	0.026*	
C5	0.80202 (8)	0.6324 (2)	0.33817 (7)	0.0189 (3)	
H5A	0.8240	0.5050	0.3594	0.023*	
C6	0.70736 (8)	0.6908 (2)	0.32626 (6)	0.0162 (2)	
C7	0.64106 (8)	0.5462 (2)	0.34610 (7)	0.0184 (2)	
H7A	0.6251	0.4323	0.3113	0.022*	
H7B	0.6750	0.4914	0.3956	0.022*	
C8	0.54900 (8)	0.64688 (19)	0.34457 (6)	0.0155 (2)	
C9	0.45856 (8)	0.7725 (2)	0.41579 (6)	0.0153 (2)	
C10	0.43573 (8)	0.9695 (2)	0.40415 (6)	0.0154 (2)	
C11	0.38429 (8)	0.6706 (2)	0.43345 (6)	0.0154 (2)	
C12	0.24277 (8)	0.8087 (2)	0.45811 (6)	0.0158 (2)	
C13	0.18356 (9)	0.6400 (2)	0.44282 (7)	0.0200 (3)	
H13A	0.1934	0.5340	0.4137	0.024*	
C14	0.10982 (10)	0.6290 (2)	0.47079 (8)	0.0251 (3)	
H14A	0.0696	0.5138	0.4613	0.030*	
C15	0.09484 (9)	0.7850 (2)	0.51241 (7)	0.0261 (3)	
H15A	0.0439	0.7774	0.5308	0.031*	
C16	0.15406 (9)	0.9525 (2)	0.52726 (7)	0.0233 (3)	
H16A	0.1435	1.0593	0.5557	0.028*	
C17	0.22911 (9)	0.9648 (2)	0.50060 (6)	0.0194 (3)	
H17A	0.2704	1.0784	0.5114	0.023*	
C18	1.02279 (10)	1.0805 (3)	0.32583 (8)	0.0324 (3)	
H18A	1.0649	1.1877	0.3205	0.049*	
H18B	1.0478	0.9499	0.3177	0.049*	
H18C	1.0203	1.0846	0.3752	0.049*	
C19	0.48791 (9)	1.1329 (2)	0.38237 (7)	0.0186 (2)	
H19A	0.5565	1.1016	0.3998	0.028*	
H19B	0.4779	1.2601	0.4039	0.028*	
H19C	0.4638	1.1450	0.3291	0.028*	
C20	0.27706 (9)	1.1291 (2)	0.36089 (7)	0.0188 (2)	
H20A	0.3065	1.2511	0.3502	0.028*	
H20B	0.2270	1.1663	0.3801	0.028*	
H20C	0.2488	1.0506	0.3160	0.028*	
H1N1	0.5810 (13)	0.620 (3)	0.4501 (10)	0.031 (5)*	

### Atomic displacement parameters (Å^2^)

**Table:**

	*U*^11^	*U*^22^	*U*^33^	*U*^12^	*U*^13^	*U*^23^
S1	0.02042 (17)	0.0570 (3)	0.0355 (2)	−0.00826 (17)	0.00457 (14)	0.0233 (2)
O1	0.0176 (4)	0.0252 (5)	0.0164 (4)	0.0004 (4)	0.0046 (3)	−0.0015 (4)
O2	0.0222 (4)	0.0140 (4)	0.0196 (4)	0.0002 (4)	0.0083 (3)	0.0024 (4)
N1	0.0167 (4)	0.0162 (5)	0.0159 (4)	0.0036 (4)	0.0061 (4)	0.0030 (4)
N2	0.0169 (4)	0.0120 (5)	0.0180 (4)	0.0002 (4)	0.0074 (4)	0.0023 (4)
N3	0.0175 (4)	0.0124 (5)	0.0193 (5)	−0.0008 (4)	0.0098 (4)	0.0018 (4)
C1	0.0162 (5)	0.0194 (6)	0.0187 (5)	0.0012 (5)	0.0058 (4)	−0.0004 (5)
C2	0.0193 (5)	0.0208 (7)	0.0190 (5)	−0.0003 (5)	0.0054 (4)	0.0011 (5)
C3	0.0164 (5)	0.0305 (8)	0.0156 (5)	−0.0043 (5)	0.0049 (4)	0.0005 (5)
C4	0.0148 (5)	0.0324 (8)	0.0169 (5)	0.0016 (5)	0.0051 (4)	0.0004 (5)
C5	0.0181 (5)	0.0220 (7)	0.0163 (5)	0.0031 (5)	0.0056 (4)	−0.0004 (5)
C6	0.0166 (5)	0.0173 (6)	0.0155 (5)	−0.0002 (5)	0.0067 (4)	−0.0020 (5)
C7	0.0181 (5)	0.0166 (6)	0.0232 (6)	0.0017 (5)	0.0104 (4)	0.0003 (5)
C8	0.0155 (5)	0.0132 (6)	0.0188 (5)	−0.0020 (4)	0.0071 (4)	−0.0012 (5)
C9	0.0166 (5)	0.0157 (6)	0.0147 (5)	0.0007 (4)	0.0068 (4)	0.0007 (5)
C10	0.0161 (5)	0.0168 (6)	0.0138 (5)	−0.0003 (4)	0.0058 (4)	−0.0001 (5)
C11	0.0167 (5)	0.0155 (6)	0.0135 (5)	0.0004 (4)	0.0050 (4)	−0.0002 (4)
C12	0.0153 (5)	0.0188 (6)	0.0140 (5)	0.0006 (5)	0.0060 (4)	0.0021 (5)
C13	0.0210 (5)	0.0186 (6)	0.0218 (6)	−0.0016 (5)	0.0092 (4)	−0.0007 (5)
C14	0.0227 (6)	0.0248 (7)	0.0309 (7)	−0.0054 (5)	0.0133 (5)	0.0023 (6)
C15	0.0232 (6)	0.0341 (8)	0.0258 (6)	−0.0004 (6)	0.0147 (5)	0.0036 (6)
C16	0.0245 (6)	0.0299 (8)	0.0180 (5)	0.0018 (6)	0.0108 (5)	−0.0033 (5)
C17	0.0193 (5)	0.0220 (7)	0.0167 (5)	−0.0014 (5)	0.0060 (4)	−0.0024 (5)
C18	0.0195 (6)	0.0420 (10)	0.0339 (7)	−0.0062 (6)	0.0071 (5)	0.0014 (7)
C19	0.0197 (5)	0.0159 (6)	0.0227 (6)	−0.0010 (5)	0.0105 (4)	0.0020 (5)
C20	0.0191 (5)	0.0170 (6)	0.0199 (5)	0.0021 (5)	0.0063 (4)	0.0036 (5)

### Geometric parameters (Å, º)

**Table:**

S1—C3	1.7679 (14)	C7—H7B	0.9900
S1—C18	1.7844 (14)	C9—C10	1.3534 (18)
O1—C8	1.2251 (14)	C9—C11	1.4413 (16)
O2—C11	1.2432 (15)	C10—C19	1.4843 (17)
N1—C8	1.3617 (15)	C12—C17	1.3898 (18)
N1—C9	1.4115 (15)	C12—C13	1.3936 (18)
N1—H1N1	0.878 (19)	C13—C14	1.3933 (17)
N2—C10	1.3893 (14)	C13—H13A	0.9500
N2—N3	1.4112 (14)	C14—C15	1.386 (2)
N2—C20	1.4690 (15)	C14—H14A	0.9500
N3—C11	1.3867 (16)	C15—C16	1.387 (2)
N3—C12	1.4194 (14)	C15—H15A	0.9500
C1—C2	1.3892 (18)	C16—C17	1.3946 (17)
C1—C6	1.3985 (18)	C16—H16A	0.9500
C1—H1A	0.9500	C17—H17A	0.9500
C2—C3	1.3952 (17)	C18—H18A	0.9800
C2—H2A	0.9500	C18—H18B	0.9800
C3—C4	1.399 (2)	C18—H18C	0.9800
C4—C5	1.3919 (18)	C19—H19A	0.9800
C4—H4A	0.9500	C19—H19B	0.9800
C5—C6	1.4024 (16)	C19—H19C	0.9800
C5—H5A	0.9500	C20—H20A	0.9800
C6—C7	1.5252 (17)	C20—H20B	0.9800
C7—C8	1.5187 (16)	C20—H20C	0.9800
C7—H7A	0.9900		
			
C3—S1—C18	103.92 (7)	C9—C10—C19	129.15 (11)
C8—N1—C9	120.41 (10)	N2—C10—C19	120.82 (11)
C8—N1—H1N1	120.2 (12)	O2—C11—N3	124.27 (11)
C9—N1—H1N1	118.4 (12)	O2—C11—C9	131.34 (11)
C10—N2—N3	105.49 (10)	N3—C11—C9	104.36 (10)
C10—N2—C20	118.41 (10)	C17—C12—C13	120.95 (11)
N3—N2—C20	113.73 (9)	C17—C12—N3	120.44 (11)
C11—N3—N2	110.70 (9)	C13—C12—N3	118.60 (11)
C11—N3—C12	126.05 (11)	C14—C13—C12	119.11 (13)
N2—N3—C12	119.72 (10)	C14—C13—H13A	120.4
C2—C1—C6	121.05 (11)	C12—C13—H13A	120.4
C2—C1—H1A	119.5	C15—C14—C13	120.34 (13)
C6—C1—H1A	119.5	C15—C14—H14A	119.8
C1—C2—C3	120.65 (13)	C13—C14—H14A	119.8
C1—C2—H2A	119.7	C14—C15—C16	120.13 (12)
C3—C2—H2A	119.7	C14—C15—H15A	119.9
C2—C3—C4	118.86 (12)	C16—C15—H15A	119.9
C2—C3—S1	116.94 (11)	C15—C16—C17	120.30 (13)
C4—C3—S1	124.14 (9)	C15—C16—H16A	119.8
C5—C4—C3	120.31 (11)	C17—C16—H16A	119.8
C5—C4—H4A	119.8	C12—C17—C16	119.15 (13)
C3—C4—H4A	119.8	C12—C17—H17A	120.4
C4—C5—C6	121.08 (13)	C16—C17—H17A	120.4
C4—C5—H5A	119.5	S1—C18—H18A	109.5
C6—C5—H5A	119.5	S1—C18—H18B	109.5
C1—C6—C5	118.04 (11)	H18A—C18—H18B	109.5
C1—C6—C7	122.72 (10)	S1—C18—H18C	109.5
C5—C6—C7	119.23 (12)	H18A—C18—H18C	109.5
C8—C7—C6	112.33 (11)	H18B—C18—H18C	109.5
C8—C7—H7A	109.1	C10—C19—H19A	109.5
C6—C7—H7A	109.1	C10—C19—H19B	109.5
C8—C7—H7B	109.1	H19A—C19—H19B	109.5
C6—C7—H7B	109.1	C10—C19—H19C	109.5
H7A—C7—H7B	107.9	H19A—C19—H19C	109.5
O1—C8—N1	122.58 (11)	H19B—C19—H19C	109.5
O1—C8—C7	121.68 (11)	N2—C20—H20A	109.5
N1—C8—C7	115.70 (10)	N2—C20—H20B	109.5
C10—C9—N1	126.34 (11)	H20A—C20—H20B	109.5
C10—C9—C11	109.09 (10)	N2—C20—H20C	109.5
N1—C9—C11	124.57 (11)	H20A—C20—H20C	109.5
C9—C10—N2	110.03 (11)	H20B—C20—H20C	109.5
			
C10—N2—N3—C11	6.12 (12)	N1—C9—C10—C19	1.3 (2)
C20—N2—N3—C11	137.50 (10)	C11—C9—C10—C19	−177.91 (11)
C10—N2—N3—C12	166.16 (10)	N3—N2—C10—C9	−4.81 (12)
C20—N2—N3—C12	−62.45 (13)	C20—N2—C10—C9	−133.47 (11)
C6—C1—C2—C3	0.30 (19)	N3—N2—C10—C19	175.01 (10)
C1—C2—C3—C4	0.31 (19)	C20—N2—C10—C19	46.35 (16)
C1—C2—C3—S1	177.43 (10)	N2—N3—C11—O2	173.15 (11)
C18—S1—C3—C2	146.76 (11)	C12—N3—C11—O2	14.66 (19)
C18—S1—C3—C4	−36.29 (13)	N2—N3—C11—C9	−4.94 (12)
C2—C3—C4—C5	−0.98 (19)	C12—N3—C11—C9	−163.44 (11)
S1—C3—C4—C5	−177.88 (10)	C10—C9—C11—O2	−176.02 (12)
C3—C4—C5—C6	1.06 (19)	N1—C9—C11—O2	4.7 (2)
C2—C1—C6—C5	−0.24 (18)	C10—C9—C11—N3	1.88 (13)
C2—C1—C6—C7	−179.19 (11)	N1—C9—C11—N3	−177.38 (10)
C4—C5—C6—C1	−0.43 (18)	C11—N3—C12—C17	130.20 (13)
C4—C5—C6—C7	178.55 (11)	N2—N3—C12—C17	−26.55 (16)
C1—C6—C7—C8	−12.81 (16)	C11—N3—C12—C13	−50.36 (17)
C5—C6—C7—C8	168.25 (11)	N2—N3—C12—C13	152.89 (11)
C9—N1—C8—O1	1.55 (19)	C17—C12—C13—C14	−0.03 (19)
C9—N1—C8—C7	179.33 (11)	N3—C12—C13—C14	−179.46 (11)
C6—C7—C8—O1	69.15 (15)	C12—C13—C14—C15	0.9 (2)
C6—C7—C8—N1	−108.66 (12)	C13—C14—C15—C16	−0.8 (2)
C8—N1—C9—C10	−72.32 (17)	C14—C15—C16—C17	−0.1 (2)
C8—N1—C9—C11	106.81 (14)	C13—C12—C17—C16	−0.90 (18)
N1—C9—C10—N2	−178.87 (10)	N3—C12—C17—C16	178.52 (11)
C11—C9—C10—N2	1.88 (13)	C15—C16—C17—C12	0.99 (19)

### Hydrogen-bond geometry (Å, º)

**Table:**

*D*—H···*A*	*D*—H	H···*A*	*D*···*A*	*D*—H···*A*
N1—H1*N*1···O2^i^	0.880 (19)	1.956 (19)	2.7816 (14)	155.7 (19)
C1—H1*A*···O1	0.95	2.38	3.0185 (16)	124
C1—H1*A*···O1^ii^	0.95	2.51	3.2342 (16)	133
C7—H7*A*···O1^iii^	0.99	2.57	3.4960 (16)	155
C19—H19*B*···O2^iv^	0.98	2.55	3.4470 (17)	152

Symmetry codes: (i) −*x*+1, −*y*+1, −*z*+1; (ii) −*x*+1, *y*+1/2, −*z*+1/2; (iii) −*x*+1, *y*−1/2, −*z*+1/2; (iv) *x*, *y*+1, *z*.
